# Tracing genomic variations in two highly virulent *Yersinia enterocolitica* strains with unequal ability to compete for host colonization

**DOI:** 10.1186/1471-2164-13-467

**Published:** 2012-09-11

**Authors:** Debora Garzetti, Hicham Bouabe, Juergen Heesemann, Alexander Rakin

**Affiliations:** 1 Max von Pettenkofer-Institute, LMU, Munich, Germany; 2 Current address: Laboratory of Lymphocyte Signalling and Development, The Babraham Institute, Cambridge, UK

**Keywords:** *Yersinia enterocolitica*, Hyper-virulent, Genome comparison, Diversity, Host colonization, Virulence factors, YscP, YopM

## Abstract

**Background:**

*Yersinia enterocolitica* is a gastrointestinal foodborne pathogen found worldwide and which especially affects infants and young children. While different bioserotypes have been associated with varying pathogenicity, research on *Y. enterocolitica* is mainly conducted on the highly virulent mouse-lethal strains of biotype 1B and serotype O:8. We demonstrate here that two *Y. enterocolitica* bioserotype 1B/O:8 strains, 8081 and WA-314, display different virulence and fitness properties in a mouse model. *In vivo* co-infection experiments revealed that strain WA-314 overcomes strain 8081 in the colonization of spleen and liver. To trace the reasons of this incongruity, we present here the first high-quality sequence of the whole genome of strain WA-314 and compare it to the published genome of strain 8081.

**Results:**

Regions previously accepted as unique to strain 8081, like the YAPI and YGI-3 genomic islands, are absent from strain WA-314, confirming their strain-specificity. On the other hand, some fitness- and bacterial competition-associated features, such as a putative colicin cluster and a xenobiotic-acyltransferase-encoding gene, are unique to strain WA-314. Additional acquisitions of strain WA-314 are seven prophage-like regions. One of these prophages, the 28-kb P4-like prophage YWA-4, encodes a PilV-like protein that may be used for adhesion to and invasion of the intestinal cells. Furthermore, a putative autotransporter and two type 1 fimbrial proteins of strain WA-314 show a sequence similarity <50% with the orthologous proteins in strain 8081. The dissimilar sequences of these proteins indicate possible different functions or interaction modes, reflecting the specific adhesion properties of *Y. enterocolitica* strains 8081 and WA-314 and thus the different efficiency of host colonization. Further important differences were found in two pYV plasmid-encoded virulence factors, YopM and YscP. The impact of these differences on virulence is discussed.

**Conclusions:**

Our study emphasizes that the virulence of pathogens can be increased, by acquiring new genes and/or improving the function of essential virulence proteins, resulting in permanently hyper-virulent strains. This work also highlights the importance of addressing genetic and phenotypic variations among closely related bacterial strains, even those belonging to the same bioserotype.

## Background

*Yersinia enterocolitica* is a globally disseminated gastrointestinal pathogen which is transmitted by the fecal-oral route, through ingestion of contaminated food or water [1]. Human clinical infections most commonly occur in young individuals and are associated with acute diarrhoea, terminal ileitis, mesenteric lymphadenitis and pseudo-appendicitis [2]. Critical elements for pathogenesis are the high pathogenicity island (HPI), carrying the siderophore-mediated iron uptake system named yersiniabactin, and the virulence plasmid pYV. While the HPI and pYV are absent in avirulent strains, they are both conserved among highly virulent strains of the three pathogenic *Yersinia* species, *Y. pestis*, *Y. pseudotuberculosis* and *Y. enterocolitica*. The 70-kb plasmid encodes a type III secretion system (T3SS) and a set of Yop effector proteins which, after injection by the T3SS into host cells, inhibit several host immune mechanisms which enable the bacteria to survive in the host environment [3].

*Y. enterocolitica* strains are heterogeneous and are classified into 6 biotypes (1A, 1B, 2, 3, 4 and 5) according to biochemical properties [4]. Biotype 1A strains, lacking both HPI and pYV plasmid, are considered as non-virulent in mice, whereas biotypes 2 to 5, which lack HPI, are low virulence (unable to kill mice) [5]. These five biotypes belong to the *Y. enterocolitica* subsp. *palearctica* and are generally isolated in Europe and Japan (termed “Old World” strains). Biotype 1B (subsp. *enterocolitica*), harboring both HPI and pYV plasmid, is highly virulent (mouse-lethal) and predominant in North America (the so called “New World” strains) [6]. More than 70 serotypes of *Y. enterocolitica* have been described; however only few of them are virulent with serotypes O:3, O:5,27, O:8, O:9, O:20 and O:13 being the most pathogenic to humans [2]. In the past, *Y. enterocolitica* bioserotype 1B/O:8 strains were predominant in the United States [7]. Nowadays these strains are also isolated in other countries; nevertheless, bioserotype 4/O:3 strains are the most commonly *Y. enterocolitica* strains found over the world [8], [9].

Many *Yersinia* research laboratories use in their studies two mouse-virulent *Y. enterocolitica* 1B/O:8 strains, named 8081 [10] and WA-314 [5]. *Y. enterocolitica* strain 8081 is an American isolate from a fatal-septicemia patient [10] and has been widely used in murine infection models. *Y. enterocolitica* strain WA-314 was isolated from the blood of a human patient and proved to be highly virulent for mice and rats [11]. Recently, in an attempt to identify chromosomal variations underlying the different properties of high versus low virulence strains, the genome of the highly virulent strain 8081 [12] has been compared to genomes of low virulence strains [13,14]. However, a complete comparative characterization of highly virulent 1B/O:8 strains with each other had not been performed. In this context, the sequence of the pYV_WA-314_ has been recently determined, showing global similarity to pYV plasmids of other *Y. enterocolitica* strains but with noticeable differences in the amino acid sequences of the T3SS proteins SycH, YopM, LcrV and YscP [15]. Phenotypic differences in autoagglutination experiments [15] and results from our pilot experiments with strains 8081 and WA-314, displaying differences in bacterial growth curves and optical density-cellular mass correlation *in vitro*, suggested that these two strains possessed distinct virulence and colonization properties.

In this work, we demonstrated that strain WA-314 overcame strain 8081 in the colonization of spleen and liver during *in vivo* co-infection experiments. Genome comparison between the genomic sequence of strain 8081 [12] and a high-quality whole-genome sequence of strain WA-314, presented in this study, allowed the identification of putative virulence factors which may account for the different *in vivo* phenotypic behavior of these two 1B/O:8 strains.

## Results

### Comparison of the infection ability of *Y. enterocolitica* strain 8081 versus strain WA-314 in mouse model

#### Y. enterocolitica strain WA-314 is a hyper-virulent strain with increased colonization in mice

**Figure 1:**
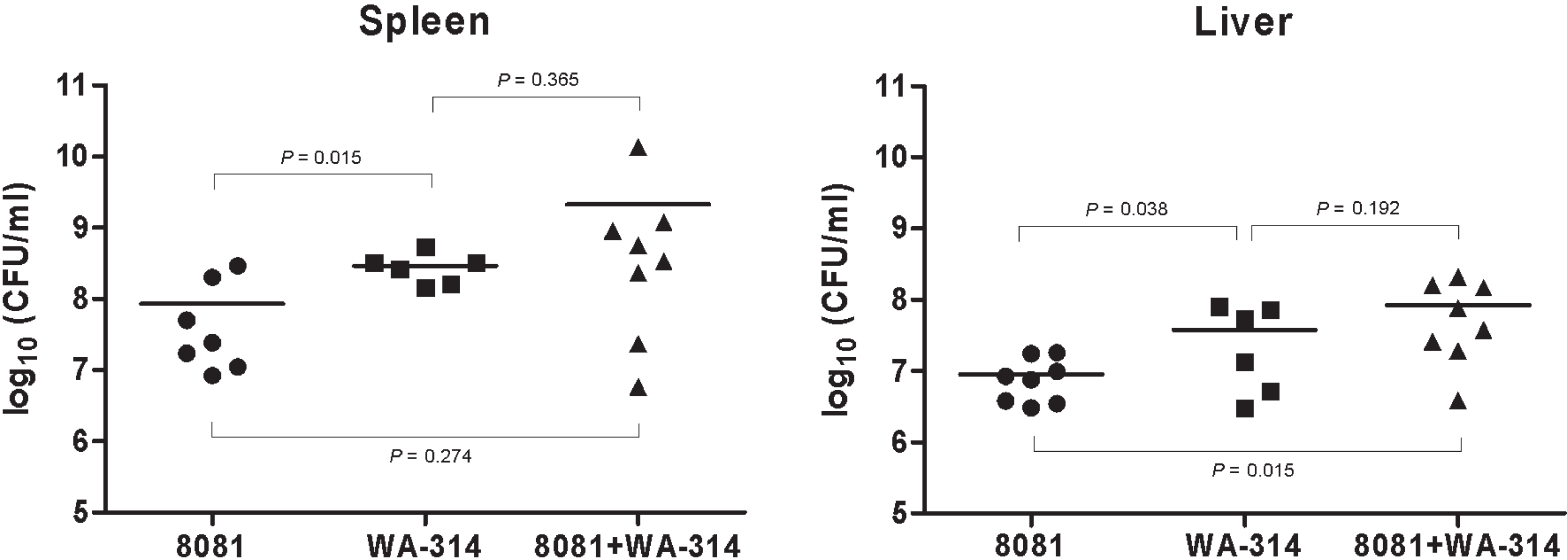
** Mouse infection experiments.** Bacterial counts in spleens and livers of mice infected with *Y. enterocolitica* strain 8081, *Y. enterocolitica* strain WA-314 and of co-infected mice with both strains are indicated. The means log_10_(CFU/ml) are displayed as error bars.

In order to directly compare the virulence efficiency of *Y. enterocolitica* strain 8081 and *Y. enterocolitica* strain WA-314, we infected 3 groups of mice with the same total CFU of strain 8081, strain WA-314, or a 1:1 mix of both. Intra-peritoneal infection was used to enable accurate and controlled injection of the dose of interest into a limited number of mice. Figure 1 indicates the bacterial loads in spleens and livers of the 3 groups of mice. Mice infected with strain 8081 contained significantly lower bacterial loads than mice infected with strain WA-314, both in the spleens (*P* = 0.01) and in the livers (*P* = 0.04). Mice co-infected with 1:1 mix of *Y. enterocolitica* strain 8081 and *Y. enterocolitica* strain WA-314 contained significantly more bacteria in liver samples, as compared to the livers of mice infected with strain 8081 alone (*P* = 0.01). However, no significant difference was shown between bacterial counts in livers of mice infected with strain WA-314 alone and with 1:1 mix of both strains 8081 and WA-314 (Figure 1). In addition, bacterial colonization in spleens of mice infected with individual versus combined strains was not significantly different.

#### Digoxigenin-labeled probes specifically differentiate between Y. enterocolitica strain 8081 and Y. enterocolitica strain WA-314

Two strain-specific digoxigenin-labeled probes were designed for differentiating *Y. enterocolitica* 8081 and WA-314 (see Methods). The strain 8081-specific probe targets the putative hemolysin gene in the YAPI region, while the probe specific for strain WA-314 targets a region inside the colicin operon specifically acquired by strain WA-314 (see below). The two probes were tested on plates with cultivated single-strain and mixed-strain colonies. Two membranes were placed sequentially on each plate and then hybridized with probes Hem_8081 and Col_WA, respectively. As expected, colonies on membranes derived from strain 8081-plates were all detected by probe Hem_8081, whereas probe Col_WA gave no signal. Probe Hem_8081 did not detect any colonies on membranes replicated from strain WA-314-plates, while WA-314 colonies were all recognized by probe Col_WA. On mixed-strain membranes, probes Hem_8081 and Col_WA detected different colonies, all colonies gave a signal with the respective probe and no colonies were recognized by both probes, indicating sensitivity and specificity of the developed test (see Additional file 1).

#### Y. enterocolitica strain WA-314 overcomes 8081 in co-infection experiments

**Figure 2:**
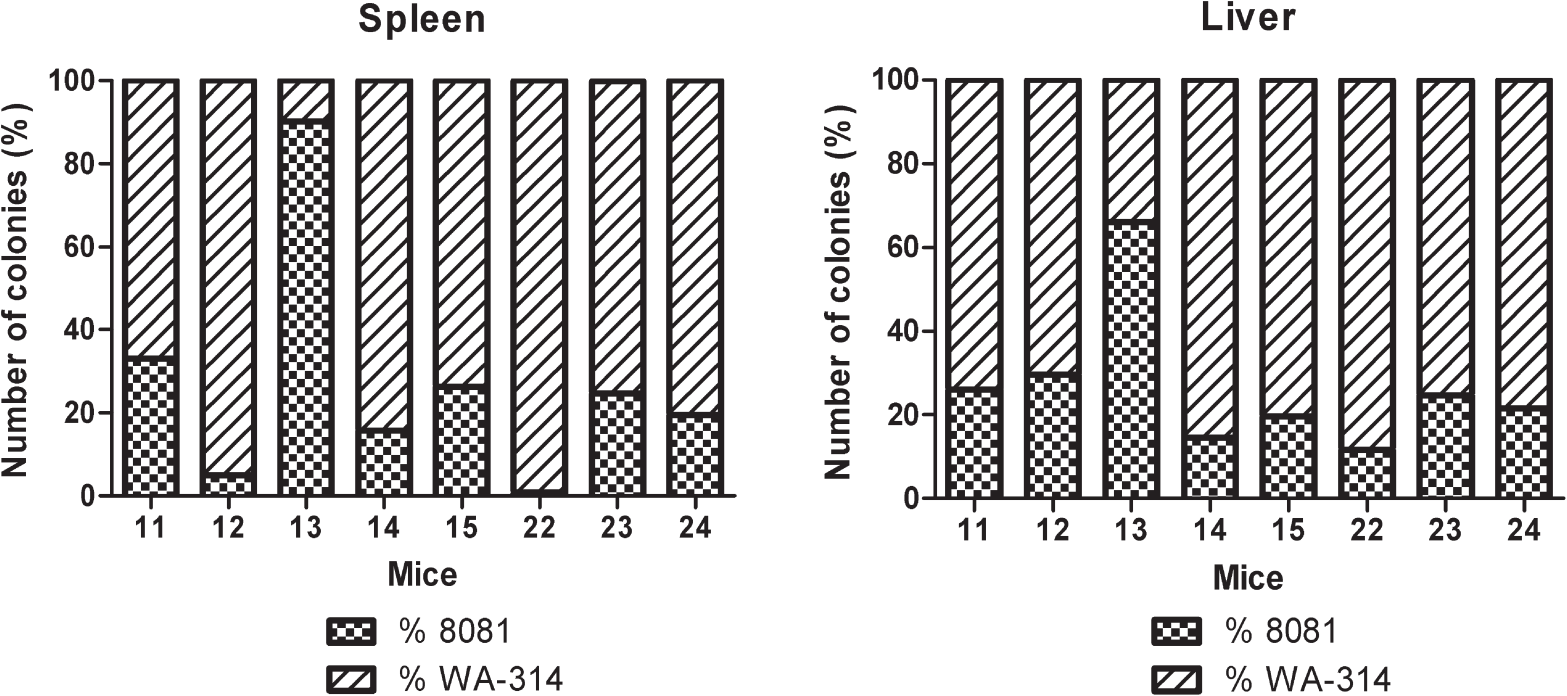
** Bacterial strain identification from co-infected mice.** Results are obtained with the colony hybridization experiments from spleens and livers of the 8 co-infected mice. *Y. enterocolitica* strain WA-314 is more abundant than *Y. enterocolitica* strain 8081 in 7 mice out of 8.

To investigate whether *Y. enterocolitica* strains 8081 and WA-314 equally colonize the organs of co-infected mice, we applied the optimized colony hybridization experiment (see Methods) on bacteria extracted from both spleens and livers (Figure 2). *Y. enterocolitica* strain WA-314, on average, accounted for 80% of the total *Yersinia* loads in spleens and livers of the co-infected mice. Only in mouse number 13 strain 8081 surpassed strain WA-314. However, the total bacterial count in this mouse’s spleen was the lowest in this study (5.81 x 10^6^), suggesting that inoculation was not correctly performed. From our own experience, there is one strongly deviating result within a group of ten mice. For these reasons, mouse number 13 was not taken into account for statistical analysis. Thus, the results of the co-infection experiments clearly indicated that strain WA-314 could out-compete strain 8081 for colonization of host organs.

### Whole genome comparison of two *Y. enterocolitica* 1B/O:8 strains

#### General features

We present here the first whole-genome high-quality sequence of *Y. enterocolitica* strain WA-314 and compare it to the published genome of *Y. enterocolitica* strain 8081 [12] (Table 1). Genome sequencing and assembly of the 129 contigs of strain WA-314 genome resulted in 4 large scaffolds and 8 contigs, with an average G + C content of 47.78%; one additional scaffold constituted the pYV plasmid (see Additional file 2). The genomes of the two *Y. enterocolitica* 1B strains share 3,736 orthologous coding sequences (CDSs). The *Yersinia* Genomic Islands 1 and 2 (YGI-1 and YGI-2) are conserved, as are the virulence-associated determinants *ail*, *inv*, the LPS operon, the flagella and urease clusters, the two General Secretion Pathways Yts1 and Yts2 and the chromosomal T3SS Ysa (Table 2). 

**Table 1:** **Genome properties of the chromosomes of*Y. enterocolitica*strains WA-314 and 8081**

**Property**	***Y. enterocolitica*WA-314**	***Y. enterocolitica*8081[**[12]**]**
Genome size	~ 4,459,631	4,615,899
G + C content	47.78%	47.27%
Number of CDSs	> 4,045	4,037
tRNA	≥ 65	81
Prophage regions	7	4

**Table 2:** **Established and putative virulence determinants of*Y. enterocolitica***

**Genomic origin**	**Protein**	**Function**	**Sequence similarity**
**Virulence-associated determinants**			
**Plasmid (pYV)**			
*yop*	Yops	Anti-phagocytic action	YopM: 75% (protein)
*ysc*	Ysc	Deliver of Yops	YscP: 72% (protein)
*yadA*	YadA	Attachment, invasion	97% (protein)
**Chromosome**			
*inv*	Invasin	Attachment, invasion	100% (protein)
*ail*	Ail	Attachment, invasion	96% (protein)
*myfEFABC*	Myf fibrillae	Intestinal colonization	97% (DNA)
HPI	Yersiniabactin	Dissemination in the host	98% (DNA)
*ureABC*	Urease	Bacterial survival in acidic environments	100% (protein)
*flhDC*	Flagella	Migration and adherence to host cells	100% (protein)
Ysa (PZ)	T3SS	Secretion of Ysps proteins	98% (DNA)
Yts1 (PZ)	GSP	Dissemination into deep tissues	99% (DNA)
Yts2	GSP	Unclear	98% (DNA)
*ystA*	Enterotoxin	Fluid loss and diarrhea	100% (protein)
**Putative virulence determinants identified in this study**			
**Chromosome**			
YE0694	Putative adhesin	Adherence	75% (protein)
YE3700	Outer membrane protein/ Autotransporter	Adherence and invasion	23% (protein)
YE1111	Fimbrial protein	Adherence	40% (protein)

Names and locus tags refer to the genome of *Y. enterocolitica* strain 8081 [12].

#### Mobile elements

**Figure 3:**
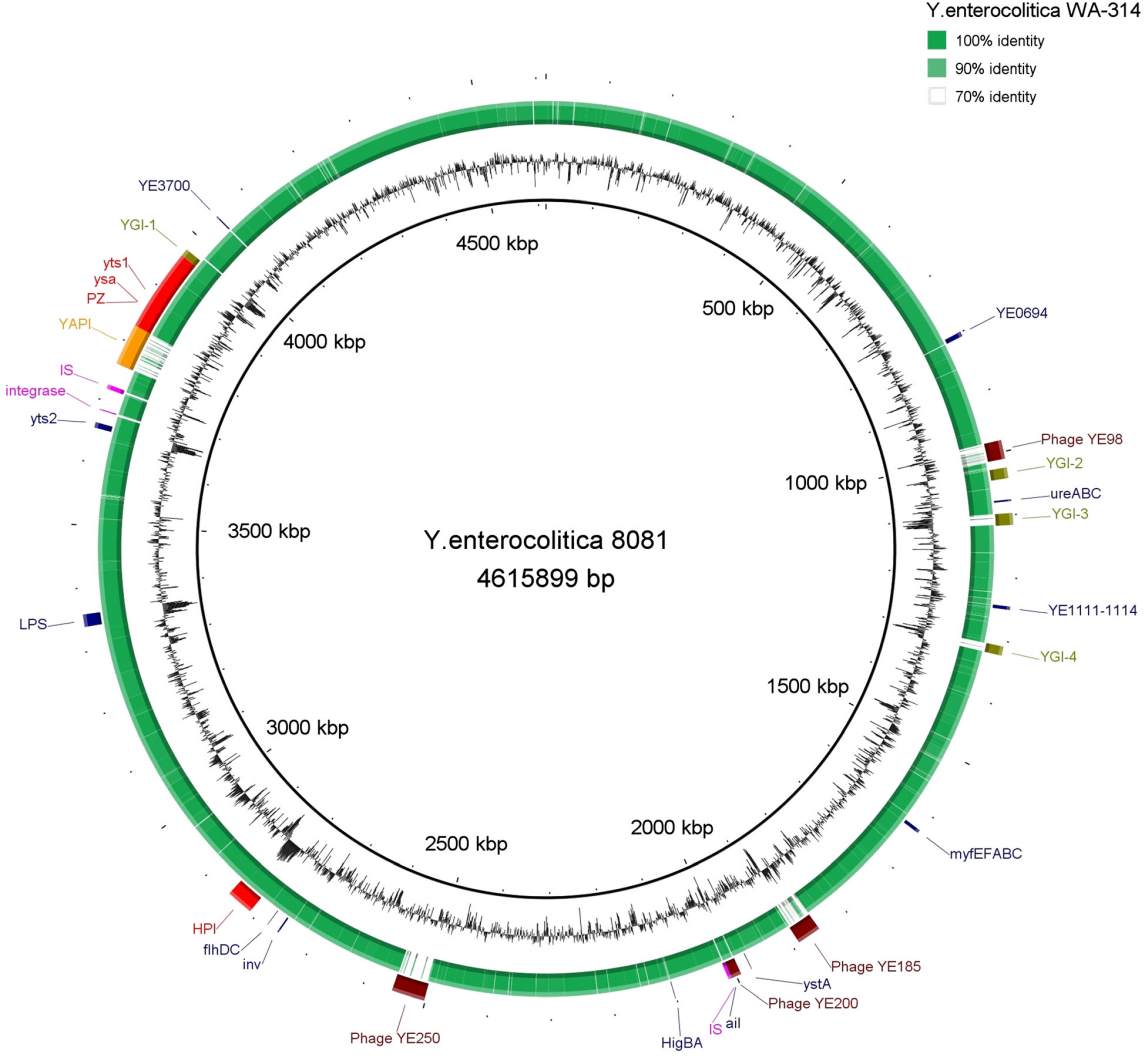
** Genomic DNA comparison.** Circular representation of the genome comparison between *Y. enterocolitica* strains 8081 and WA-314, obtained with BRIG [41]. G + C content of the reference strain (8081) is also shown. The position of significant regions and virulence genes is marked in the outer ring.

The largest source of genetic variation between strains 8081 and WA-314 is represented by genomic islands and prophages (Figure 3 and Table 3). In particular, the YAPI (*Yersinia* Adhesion Pathogenicity Island) and the YGI-4 are absent from *Y. enterocolitica* strain WA-314. The YGI-4 of strain 8081 is a putative integrated plasmid variably present in other *Y. enterocolitica* 1B strains, whereas the virulence-associated YAPI is a 66-kb region located within the Plasticity Zone of *Y. enterocolitica* strain 8081 [12]. This pathogenicity island is integrated between an intact and a partial tRNA-Phe copy that is complete in WA-314. The YAPI carries encoded plasmid-related functions and virulence determinants such as a type IV secretion system, a putative hemolysin, a toxin-antitoxin system (CcdAB family) and an arsenic-resistance operon. Most differences between the genomes of strains WA-314 and 8081 are due to prophages or prophage remnants. We identified 7 prophage-like regions (see Additional files 3 and 4) in *Y. enterocolitica* strain WA-314, encoding numerous hypothetical proteins that may also contribute to the high virulence of this strain. Considering the prophage diversity, two of the WA-314 prophage regions, YWA-1 and YWA-2, appear to be highly similar to prophages in strain 8081, ΦYE185 and ΦYE200, and Φ98, respectively. Since these regions are not located in the same chromosomal context, they are likely strain-independent acquisitions. A 28-kb P4-like prophage in strain WA-314 (YWA-4) is located in a region corresponding to YGI-3, a 19-kb putative integrated plasmid in strain 8081. This WA-314-specific prophage encodes a PilV-like protein (locus tag: YWA314_12491), which may be a part of an ancient type IV *pil* operon, and contains a Shufflon N-terminal region highly similar to adhesion exoproteins in *Y. enterocolitica* subsp. *palearctica* and in *Y. intermedia*. It has been shown that type IV pili are used by enterobacteria during adhesion to and invasion of the human intestinal cells [16-18]. Whether the WA-314-specific PilV-like protein enhances bacterial intestinal colonization has to be addressed. 

**Table 3:** **Significant regions of difference between*Y. enterocolitica*strains 8081 and WA-314**

**Region or gene cluster**	**Size in strain 8081**	**Size in strain WA-314**	**General description**	**Comment**
YAPI (PZ)	66 kb	absent	Pathogenicity island	Absent from strain WA-314
YGI-3	19 kb	28 kb	Putative integrated plasmid in strain 8081, putative prophage in strain WA-314	Strain-specific region
YGI-4	15 kb	absent	Putative integrated plasmid	Absent from strain WA-314
YE1922	0.2 kb	absent	Toxin/Antitoxin system (HigBA)	Absent from strain WA-314
YE1923	0.3 kb	absent		
YWA314_17584-YWA314_17599	absent	4 kb	Type II restriction-modification system	Absent from strain 8081
YWA314_20244-YWA314_20259	absent	3 kb	Putative group A colicin operon	Absent from strain 8081
				Present in subsp. *palearctica*
YWA314_07469	absent	1 kb	Xenobiotic-acyltransferase	Absent from strain 8081
				Present in subsp. *palearctica*

#### Y. enterocolitica strain WA-314 specific genes

Genes specifically present in *Y. enterocolitica* strain WA-314 include an additional restriction-modification (RM) system, a four-gene putative colicin cluster and a xenobiotic-acyltransferase (XAT)-encoding gene. The RM cluster, present also in *Y. frederiksenii*, consists of 4 genes (locus tags: YWA314_17584-17599), encoding an EcoRII-like type II restriction endonuclease, a very-short-patch repair (Vsr) endonuclease, a cytosine methylase and a DNA-binding protein. Restriction modification systems defend bacteria against foreign DNA, by means of the endonuclease that recognizes non-methylated cytosines of incoming DNA and cleaves it at defined sites [19]. The host DNA is protected from cleavage as the internal cytosines are modified by the methylase activity; however, this methylation increases C to T mutations, which are in turn recognized and repaired by Vsr proteins [20]. Colicins are bacteriocins, proteins produced by bacteria which are lethal for closely related strains. Colicins are genetically organized in operons, with a variable genetic structure containing one or more genes encoding colicin, immunity, and lysis proteins [21]. The cluster found in *Y. enterocolitica* strain WA-314 genome is composed of four annotated genes (locus tags: YWA314_20244-20259), encoding two putative immunity proteins and two putative colicins. It is located near a phage anti-termination protein-encoding gene, a transposase and a pilus chaperone-encoding gene, reflecting a similar structure in other *Yersinia* species, such as *Y. enterocolitica* subsp. *palearctica* bioserotype 4/O:3 [13]. Our spot-on-lawn assay could not detect any colicin activity in *Y. enterocolitica* strain WA-314 against strain 8081 and the control strain *E. coli* K12, even after mitomycin C induction (data not shown). Thus, the colicin operon seems to be not active under the tested *in vitro* conditions. The XAT protein (locus tag: YWA314_07469) belongs to a family of resistance enzymes that catalyze the acetylation of a variety of hydroxyl-bearing acceptors such as chloramphenicol and streptogramin. It may be implicated in the inactivation of xenobiotics, leading to resistance.

The insecticidal toxin cluster, described in biotype 2–5 strains, was not found in the sequence of *Y. enterocolitica* strain WA-314 genome, in contrast to previously reported experimental data [22].

#### Newly identified potential virulence genes

*Y. enterocolitica* strain 8081 harbors a 635-aa outer membrane protein (locus tag: YE3700), which has only 23% of sequence similarity with its orthologous in strain WA-314, a 902-aa putative autotransporter (locus tag: YWA314_14949). These two proteins are situated in the same genomic region, indicating a common chromosomal origin and subsequent mutations during the evolution of the two strains. Interestingly, both genes have a low G + C content: 43.1% in strain 8081 and 39.2% in strain WA-314. Autotransporters are known virulence factors in Gram-negative bacteria, as they mediate bacterial aggregation and biofilm formation, as well as adhesion and invasion of epithelial cells. All classical autotransporters share a common organization: a signal peptide followed by an N-terminal passenger domain and a C-terminal translocator domain, with the passenger domain being involved in pathogenesis [23]. The protein encoded by strain 8081 has an autotransporter beta-domain at the C-terminus, but no known domains at the N-terminus. The protein of strain WA-314 contains a pertactin-like passenger domain at the central region and an autotransporter beta-domain at the C-terminus, a typical organization found in the homologous AidA-I protein in *Escherichia coli*[24]. Both proteins carry no signal peptide, according to *in silico* prediction algorithms (SignalP, version 4.0 [25]). However, as signal peptides have no high sequence homology among autotransporters [26], the SignalP program may not recognize the presence of signal peptides in the two analyzed amino acid sequences. The dissimilar sequences of the passenger domain of these two proteins may be responsible for the specific adhesion properties of *Y. enterocolitica* strains 8081 and WA-314. Further support for this possibility comes from another putative adhesin (locus tag: YE0694) in strain 8081, which is only 75% identical to the homologous protein in strain WA-314 (locus tag: YWA314_00878).

Fimbriae (or pili) are biological structures involved in adherence to host epithelial cells and important virulence factors for several diseases affecting the urinary, genital and gastrointestinal tracts [27]. A type 1 fimbrial operon was identified in *Y. enterocolitica* strain 8081 as region of difference among the *Yersinia* species [12]. The DNA sequence of this operon is 84% similar to the operon in strain WA-314. In addition, two fimbrial proteins in strain 8081 (locus tags: YE1111 and YE1114) show only 40% of sequence similarity with the orthologous proteins in strain WA-314 (locus tags: YWA314_11901 and YWA314_11891). These proteins are absent from *Y. enterocolitica* subsp. *palearctica* strains and, therefore, can be considered specific factors for highly virulent strains.

#### pYV plasmid-encoded genes

**Figure 4:**
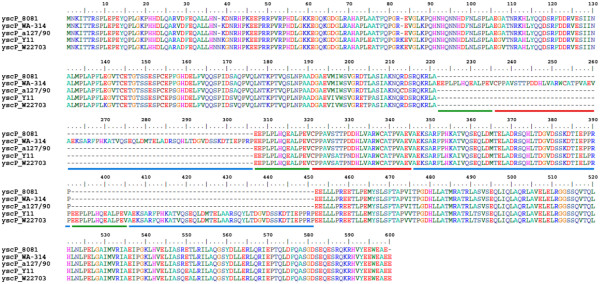
** Protein sequence alignment of YscP.** The analyzed YscP protein sequences are taken from *Y. enterocolitica* strains 8081 (O:8), WA-314 (O:8), a127/90 (O:8), Y11 (O:3) and W22703 (O:9) (see Table 6 for accession numbers). The green, red and blue lines highlight the repeats of 14 aa, 25 aa and 46 aa, respectively.

**Figure 5:**
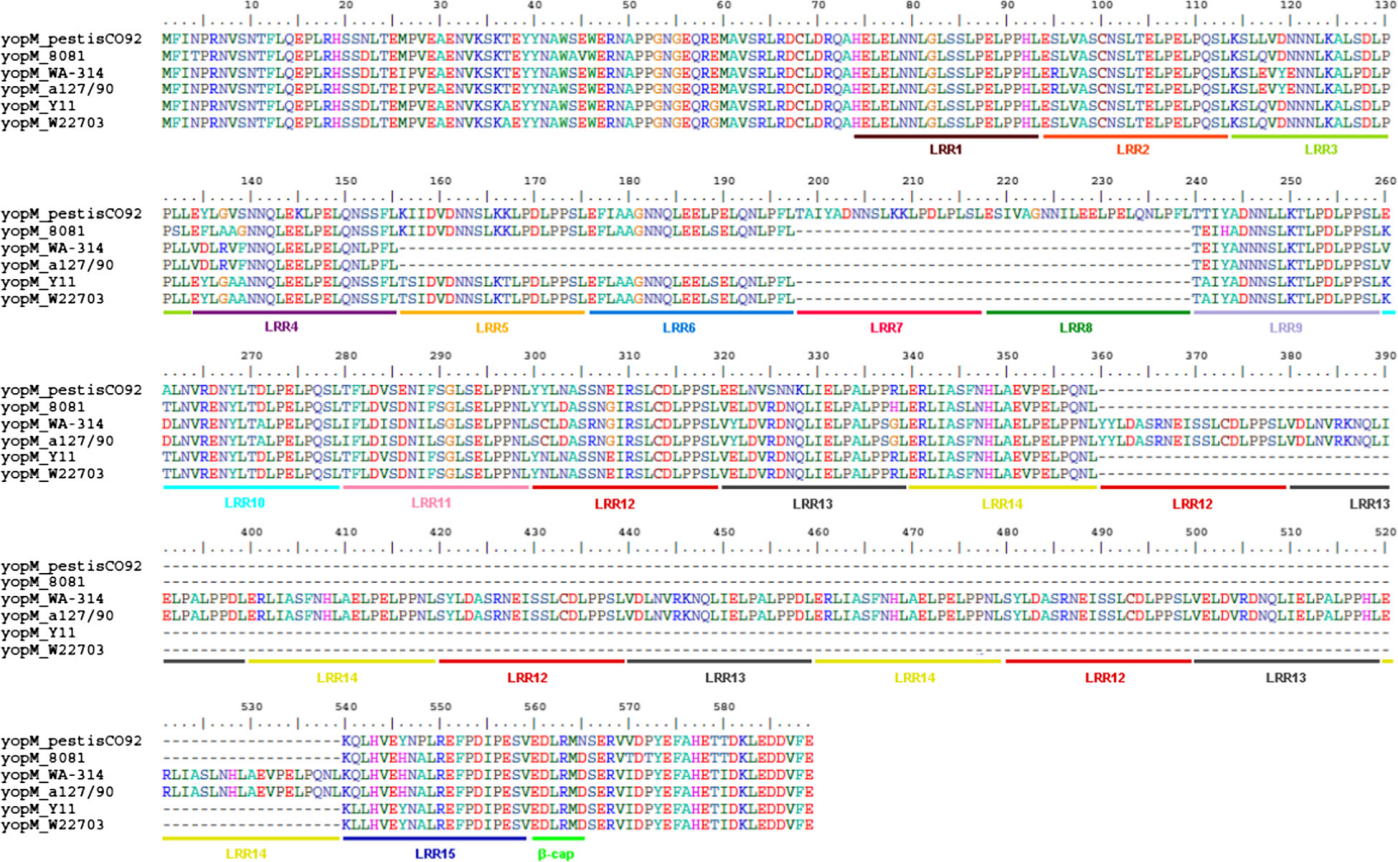
** Alignment of the sequences of YopM proteins.** Protein sequences are obtained from *Y. enterocolitica* strains 8081 (O:8), WA-314 (O:8), a127/90 (O:8), Y11 (O:3) and W22703 (O:9), and *Y. pestis* CO92, as reference strain (see Table 6 for accession numbers). LRR numbers follow the published notation [29].

The nucleotide sequence of the pYV_WA-314_ was, as expected, nearly identical to the previously published sequence [15]. The pYV_WA-314_ sequence was also compared to the pYV plasmid sequence of strain 8081. Besides two repeat regions, the main differences were found in two T3SS protein-encoding genes, *yscP* and *yopM*. The amino acid sequence of the YscP protein, a component of the T3SS injectisome which determines the length of the needle, varies within *Y. enterocolitica* species in the specific number of three repeated motifs (of 14 aa, 25 aa and 46 aa respectively) [28]. Consistent with these data, strain 8081 harbors a 1359-bp gene, encoding a 452-aa YscP protein, while in strain WA-314 *yscP* is a 1617-bp gene encoding a protein of 538 residues, with 3 additional repeats (Figure 4). Interestingly, YscP proteins from low virulence strains *Y. enterocolitica* Y11 and W22703 (both of 515 residues) possess 2 extra repeats absent from highly virulent strains of serotype O:8. The T3SS effector YopM is a leucine-rich repeat (LRR) protein of unclear function. YopM sequences from different *Yersinia* strains contain duplications and deletions in the number of LRRs, with consequent variability in the YopM length [29]. YopM from *Y. pestis* CO92, for example, has 15 LRRs, while YopM from *Y. enterocolitica* strains Y11 and W22703 have 13 LRRs. The *yopM* gene in strain 8081 encodes a protein of 367 residues with 13 LRRs, while in strain WA-314 YopM is a 505-aa protein with 24 LRRs (Figure 5). In particular, the YopM sequence from pYV_WA-314_, which is 100% identical to the YopM sequence from the O:8 strain a127/90, has 3 additional copies of LRR12-LRR13-LRR14. 

### Phylogenetic position of *Y. enterocolitica* strain WA-314

To gain insights into the evolution of *Y. enterocolitica* subspecies, we selected 9 strains representing the three *Y. enterocolitica* groups, classified according to the virulence grade: avirulent (biotype 1A, strains IP2222 and NF-O); low virulence (biotypes 3 and 4, strains Y11, Y5.27P, 105.5R(r), Y5307 and Y8265) and highly virulent (bioserotype 1B/O:8, strains 8081 and WA-314). The *Y. pestis* strain CO92 was selected as outgroup, known *a priori* to be an outlier to the ingroup sequences and chosen to root the tree.

**Figure 6:**
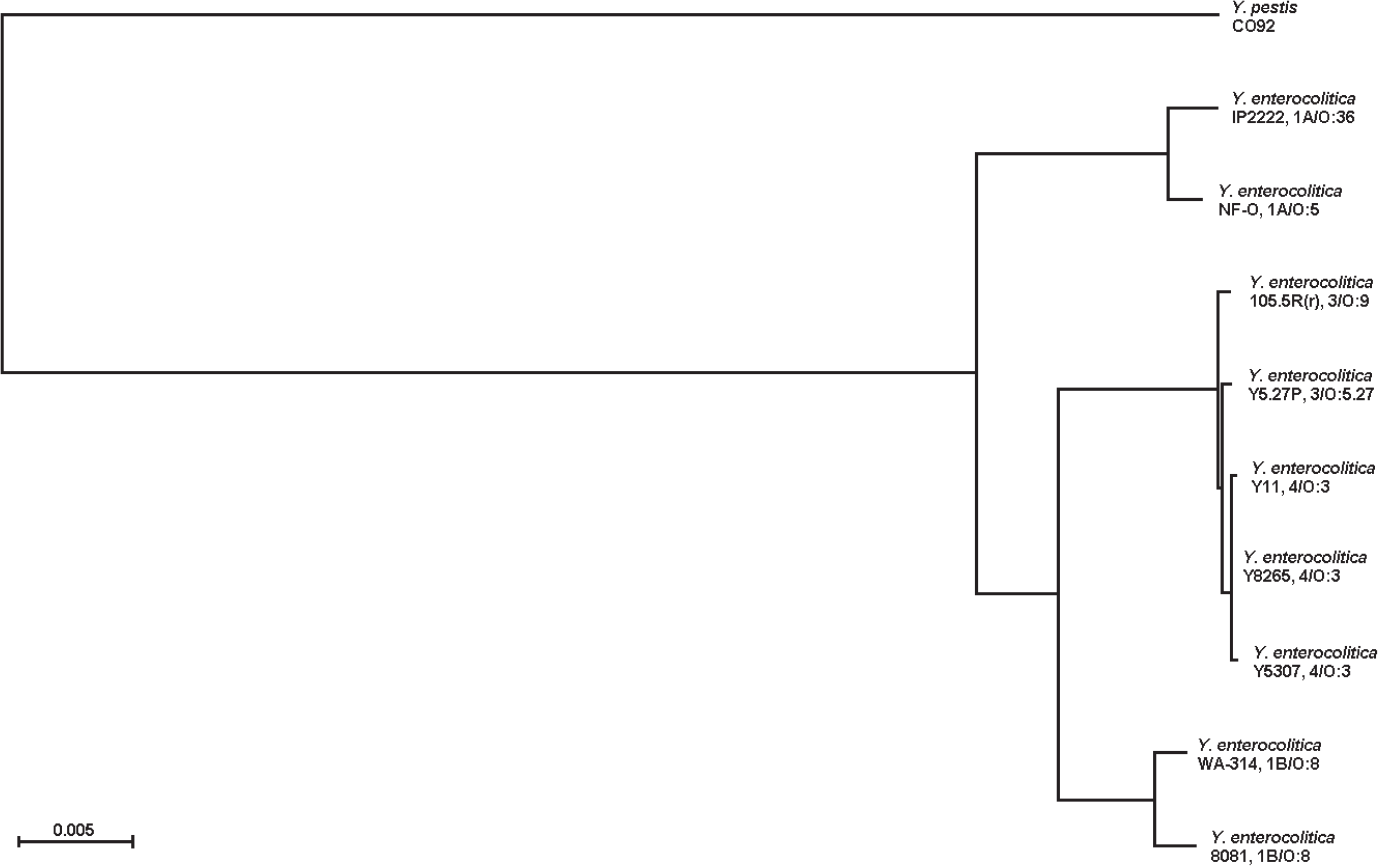
** Phylogenetic analysis of the*Yersinia enterocolitica*species.** The constructed phylogram is based on DNA sequences and rooted using the genes of *Y. pestis* CO92. Nine different *Y. enterocolitica* strains were used*:* IP2222 and NF-O (avirulent); 8081 and WA-314 (highly virulent); Y11, Y5307, Y8265, Y5.27P and 105.5R(r) (low virulence). The distance is scaled in units of the expected fraction of changed nucleotides.

The concatenated tree (Figure 6) sorts three monophyletic clusters for the three *Y. enterocolitica* groups, as expected. The intragroup genetic distances show that biotypes 3 and 4 are clustered more tightly than biotypes 1A and 1B; therefore genomes of low virulence strains have lower plasticity, when compared to avirulent and highly virulent strains, which present a higher degree of genetic variation. Concerning the evolution of *Y. enterocolitica*, this analysis indicates that the three groups evolved independently from a common ancestor, with biotype 1A strains being the first to diverge, followed by biotype 1B and 2–5 strains. According to our phylogeny reconstruction, non-pathogenic strains are evolutionarily closer to *Y. pestis* than the pathogenic *Y. enterocolitica*.

## Discussion

Highly virulent *Yersinia enterocolitica* strains have been extensively used to clarify the virulence/fitness mechanisms of this heterogeneous gastrointestinal pathogen. The use of different bioserotype 1B/O:8 strains is normally not considered a variable factor in experimental animal infection procedures. In this study, however, two *Y. enterocolitica* 1B/O:8 strains, 8081 and WA-314, demonstrated different virulence behaviors in mice, both in single strain infection and in competition assays, with strain WA-314 showing a higher virulence/fitness level. A further characterization of these two strains was therefore necessary to uncover the genetic background behind the phenotypic differences.

Genome comparison between a high-quality sequence of *Y. enterocolitica* strain WA-314 and the published sequence of *Y. enterocolitica* strain 8081 [12] revealed, besides an overall similarity in gene composition, important differences in genomic islands and prophages. In pathogenic bacteria, genomic islands and prophages represent a large source of inter- and intra-species genetic variation [30]. Pathogenicity islands also play an essential role in spreading virulence genes through lateral gene transfer, an evolutionary process by which bacterial pathogens acquire new virulence factors en bloc. Our analysis revealed that the YGI-4 and the pathogenicity island YAPI, carrying a type IV secretion system known to be involved in *Y. pseudotuberculosis* virulence [31], are absent from strain WA-314. Moreover, both 8081 and WA-314 strains harbor prophages that have no or low similarity. We observed that the mobile genetic elements tend to occupy the same positions in both 1B/O:8 genome backbones. The P4-like prophage YWA-4, for example, is inserted in strain WA-314 in the same genomic region as the YGI-3 plasmid-like element in the chromosome of strain 8081. This speaks in favor of the presence of “hot-spots” for the integration of the acquired genetic material. Such hot spots, besides being integration sites, might represent genome regions with high gene expression potential, an important factor for the homing of laterally acquired genetic clusters. Taken together, these data emphasize the important role of horizontal gene transfer and mobile genetic elements in the evolution and genetic diversification among pathogenic *Yersinia*.

Besides mobile elements, the genomes of *Y. enterocolitica* strains 8081 and WA-314 differ in a number of gene clusters and single protein-encoding genes. Strain WA-314 specific acquisitions include a XAT-encoding gene, a RM system and a putative colicin cluster. Strain 8081, on the other hand, harbors a specific toxin/antitoxin system, similar to the HigBA family. Nucleotide polymorphisms in homologous genes also contribute to the genetic variation between strains 8081 and WA-314. The plasticity of *Y. enterocolitica* 1B/O:8 genomes mirror the versatile lifestyles of this heterogeneous bacterial species, found in human, animal and environmental sources, and results from the on-going process of adaptation. By living in contact with various microbial communities in different niches, *Y. enterocolitica* experiences frequent opportunities for exchanging genetic material.

One of the main challenges of comparative genomics is to identify genes involved in pathogenesis. Virulence factors are generally involved in adherence, invasion, colonization of the host, interference with host defense mechanisms and damage to the host [32]. A putative autotransporter, an adhesin and two fimbrial proteins show low sequence similarity in the genomes of strains 8081 and WA-314. Together with a type IV pilus, present only in the genome of strain WA-314, they may contribute to adhesion and colonization of host tissues. Such new potential virulence determinants may be able to explain the phenotypic differences observed *in vivo* between *Y. enterocolitica* strains 8081 and WA-314. The real involvement of these proteins in virulence, however, needs to be elucidated.

Established virulence-associated determinants of *Y. enterocolitica* have been extensively studied and reviewed [33]. One group is pYV plasmid-encoded, such as YadA, the T3SS secretion machinery Ysc and the T3SS effectors Yops, while the other group is encoded within the chromosome, for example Inv, Ail and the HPI. The genomes of *Y. enterocolitica* strains 8081 and WA-314 show no significant differences in the sequences of these classical virulence markers, except for the plasmid-encoded effector protein YopM and YscP, the “needle ruler” of the *Yersinia* injectisome. The function of the YopM protein is not completely understood. However, it has been shown to form a complex with two intracellular serine/threonine kinases, protein kinase C-like 2 (PRK2) and ribosomal S6 protein kinase 1 (RSK1) [34]. In *Y. pseudotuberculosis*, the interaction with RSK1 requires the region from LRR12 to C-terminus of YopM [35], whereas PRK2 binding involves the LRR6 to LRR15 region of YopM [36]. Both RSK1 and PRK2 interaction domains of YopM are critical for virulence, e.g. by inducing production of IL-10, as demonstrated by different YopM mutant proteins [36]. Interestingly, *Y. enterocolitica* strain 8081 encodes a YopM of 367 residues with 13 LRRs, while YopM of strain WA-314 has 505 residues and 24 LRRs. Therefore, further studies are required to elucidate whether the different number of LRRs in YopM proteins of strain 8081 and WA-314: i) generally results in the interaction of the corresponding YopM proteins with different targets in the host, ii) especially causes distinct interaction or binding affinity with PRK2 and RSK1 and iii) has different consequences on the virulence of *Y. enterocolitica* in the mouse infection model. YscP, a protein highly variable within *Y. enterocolitica* species, determines the needle length of the *Yersinia* spp. injectisome, with a linear correlation between the size of YscP and the needle length [28]. It has been shown that the *Y. enterocolitica* needle needed to have a minimal length to be fully functional [37]. Such a minimal needle length, which also correlated with the length of the YadA adhesin, provided optimal contact between the needle and the host cell membrane. Thus shorter YscP proteins or longer YadA proteins led to suboptimal Yop translocation [37]. Curiously, YscP of *Y. enterocolitica* strain 8081 contains 452-aa, while in strain WA-314 YscP is 538-aa long. As YadA length is unchanged between *Y. enterocolitica* strains WA-314 and 8081 (as predicted by gene sequence comparison), we propose that the longer YscP protein in strain WA-314 would allow higher Yop translocation efficiency than strain 8081 and, therefore, improved virulence activity. This would partly explain the different phenotypes of strains 8081 and WA-314 observed in the co-infection experiment.

Small reproducible differences between the *in vitro* growth rates of *Y. enterocolitica* strains 8081 and WA-314 have been documented, with strain WA-314 growing slightly faster than strain 8081 (data not shown). Thus the lower *in vivo* colonization ability of strain 8081 might be related to its growth behavior and to metabolic and regulatory factors, without regard to virulence determinants. However, no obvious differences in metabolic and nutrient acquisition systems have been found between strain 8081 and WA-314 genomes. *In vitro* growth conditions for *Yersiniae* are also extremely different from those *in vivo*: for example, *Yersinia* optimal growth temperature is 27°C, in contrast to the *in vivo* temperature of 37°C, and most virulence factors are only expressed at 37°C. Thus a correlation between the *in vitro* growth rates of *Y. enterocolitica* strains 8081 and WA-314 and their *in vivo* colonization properties is unlikely, but such a possibility cannot be completely excluded.

**Figure 7:**
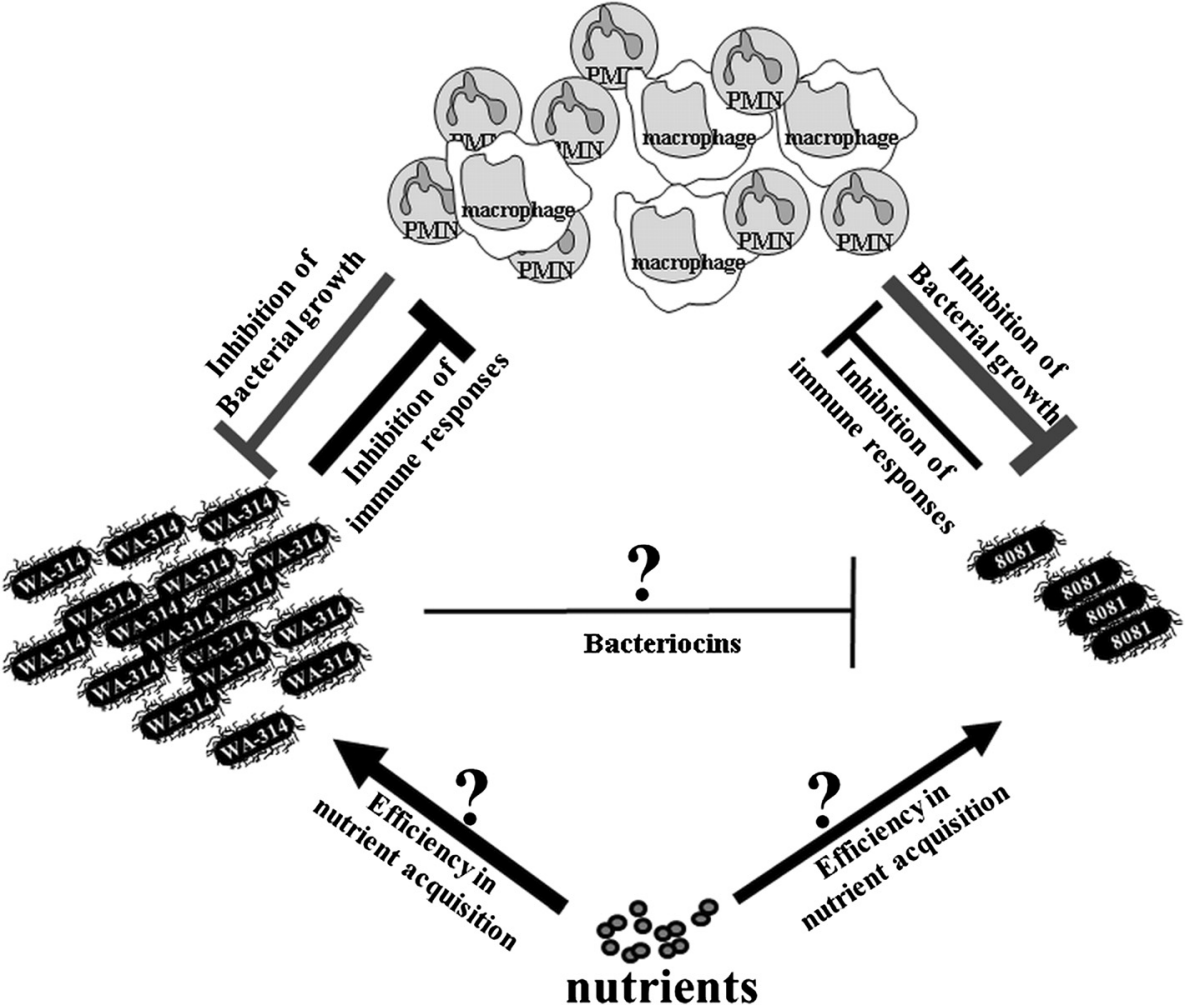
** Representation of the micro-environmental factors influencing replication and bacterial pathogenesis of*Y. enterocolitica*strains WA-314 and 8081.** Strain WA-314 appears to be better adapted than 8081 strain, probably by counteracting more efficiently the bactericidal activities of the host immune cells. The growth of strain 8081 may be also inhibited by the direct effect of bacteriocins produced by strain WA-314 bacteria. The thickness of the arrows reflects the strength and efficiency of the depicted processes.

It is well known that pathogenesis induced by bacteria is dependent on bacterial proliferation in the host. Successful invasion of and growth in host niches requires adaptation of the pathogen to the host micro-environment. Micro-environmental factors influencing bacterial colonization of the host include nutrient composition and availability in the host niches, on one hand, and the host defense mechanisms, on the other hand. In the case of co-infection or co-existence of different pathogens in the same micro-environment, a further important factor is represented by the ability of each pathogen to compete against each other for host niches and nutrient sources. Pathogens are adapted to those micro-environmental factors. They carry adhesion molecules, which enable invasion of specific tissue and cell types, and metabolic systems, such as iron-binding proteins. They also express virulence factors, which counteract immune responses of the host, and molecules, like bacteriocins, which inhibit the growth of closely related bacterial strains, thus enabling competition against other pathogens. In our co-infection model (intra-peritoneal route) with two highly virulent *Y. enterocolitica* strains, strain WA-314 seemed to be better adapted than strain 8081 to the micro-environmental factors encountered in host tissues like spleen and liver (Figure 7). Thus the present data suggest that *Y. enterocolitica* strain WA-314 is a hyper-virulent strain. In fact, mice infected with strain 8081 showed lower bacterial loads than mice infected with strain WA-314 and strain 8081 could not colonize the host as efficiently as strain WA-314, even in the absence of a competitor pathogen. The colicin operon found in strain WA-314 showed no *in vitro* activity, but we cannot exclude that this colicin could be effectively expressed *in vivo* at the host’s environmental conditions, thus probably conferring additional advantage to strain WA-314 against strain 8081. Finally, the identified putative adhesion proteins, that have been shown here to be specifically acquired by strain WA-314, may also contribute to the higher capacity of this strain to invade the host.

## Conclusions

This study demonstrated that *Y. enterocolitica* highly virulent strains exhibit significant strain-to-strain genotypic and phenotypic differences, resulting in differences in their pathogenicity. Accordingly, virulence of pathogens can be increased, e.g. by acquiring new genes and/or improving the function of essential virulence proteins. Thus, understanding the genetic factors which allow bacterial hyper-virulence would enable the design of improved therapeutic strategies against such strains.

## Methods

### Ethics statement

All animal work was performed in strict accordance with the German regulations of the Society for Laboratory Animal Science (GV-SOLAS) and the European Health Law of the Federation of Laboratory Animal Science Associations (FELASA). The protocol was approved by the Regierung von Oberbayern, Sachgebiet 54 (Verbraucherschutz und Veterinärwesen): animal licensing committee permission no. 2531–6509. All efforts were made to minimize suffering.

### Bacterial strains and growth conditions

This study was conducted with wild-type *Y. enterocolitica* strains 8081 [10] and WA-314 [11], obtained from the strain collection of our Institute. Both strains were mouse-passaged twice, to select the most virulent bacteria, and stored in 20% glycerol medium at −80°C. For mouse infection, exponential-phase bacteria were grown overnight in LB medium at 27°C, diluted 1:50 in fresh LB medium and grown for 80 min at 37°C to allow the expression of virulence factors. After pelleting and washing in Dulbecco’s Phosphate Buffered Saline (DPBS), bacteria were adjusted to the appropriate CFU/ml as infection dose.

### Mouse infection and bacterial count

Female C57/BL6 mice were individually identified with ear tags and randomly assigned to three experimental groups containing eight mice per group: groups consisted of mice to be infected with either *Y. enterocolitica* strain 8081, strain WA-314 or both in combination (Table 4). Seven-week old mice were challenged by intra-peritoneal (i.p.) injection with 0.6 ml PBS, containing 1.7 x 10^4^ bacteria for the single-strain-infection and 1.2 x 10^4^ bacteria for the co-infection (6 x 10^3^ bacteria of each strain were 1:1 mixed prior to injection). Bacterial numbers in the co-infection mix was confirmed by colony hybridization (see below). Mice were weighed every day and sacrificed 5 days post infection by inhalation of carbon dioxide. Spleens and livers were recovered and 1 ml of DPBS was added to each organ, which was homogenized with a tissue blender for 4 min. Bacterial counts were determined by plating serial dilutions of organ homogenates on LB agar followed by growth at 27°C for 20–24 hours. Differences in mice bacterial colonization were assessed by unpaired *t* tests and *P* values ≤ 0.05 were considered significant.

**Table 4:** C57/BL6 mice and injected bacteria doses used in this study

**Mouse number**	**Injected*Y. enterocolitica*strain**	**Infection dose**
1-2-3-4-5-16-17-18	8081	1.7 x 10^4^ bacteria
6-7-8-9-10-19-20-21	WA-314	1.7 x 10^4^ bacteria
11-12-13-14-15-22-23-24	8081 + WA-314	1.2 x 10^4^ bacteria

### Digoxigenin-labeled probes

We designed two digoxigenin-dUTP-labeled probes to be used in the DNA-DNA colony hybridization experiment for distinguishing *Y. enterocolitica* strain 8081 from strain WA-314, by selecting strain-specific regions in the 8081 and WA-314 genomes. The 162-bp probe specific for detecting strain 8081 targets part of the putative hemolysin gene (locus tag: YE3454) encoded by the YAPI, whereas the 234-bp probe specific for strain WA-314 targets a region inside the colicin cluster (locus tag: YWA314_20259). Blast analysis revealed that our probe sequences did not align to any genomic regions in other bacteria, thus confirming species- and strain-specificity of these probes. Both probes were generated as PCR products with digoxigenin-11-dUTP incorporated as a labeling molecule, using the PCR DIG Probe Synthesis Kit (Roche, Mannheim, Germany). Primers and PCR conditions are given in Table 5.

**Table 5:** Primers and PCR conditions for generation of the digoxigenin-labeled probes

**Primer**	**Sequence**	**Probe**	**PCR conditions**
Hem_8081_FW	5'-CAATATGACTACCGACCCGGTTAC-3'	Hem_8081	- Denaturation at 95°C for 2 min
Hem_8081_RV	5'-GGATACATCTGCTGGGCGATATAC-3'	162 bp	- 30 cycles:
			denaturation at 95°C for 30 sec
Col_WA_FW	5'-CGATCGTAGTAGTAAGGCAACTCC-3'	Col_WA	annealing at 60°C for 30 sec
Col_WA_RV	5'-GACGGTATCATGCCCATAACTG-3'	234 bp	elongation at 72°C for 40 sec
			- Final elongation at 72°C for 7 min

### Preparation of membranes for colony hybridization

Plates from overnight growth were chilled at 4°C before colony lifts. Nylon membranes (Roche) were placed onto the LB agar surface for 5 min, transferred onto new LB agar plates and incubated at 27°C for 4 h to allow further bacterial growth. Membranes were removed from the LB agar plates and placed colony-side up on filter papers soaked with 10% w/v sodium dodecyl sulphate (SDS) for 10 min. This procedure was repeated with a denaturation solution (0.5 M NaOH and 1.5 M NaCl; pH 11.5) for 15 min, a neutralization solution (1.5 M NaCl and 1.0 M Tris–HCl; pH 7.4) for 15 min and 2 X SSC (stock solution of 20 X SSC: 3 M NaCl and 0.3 M Na_3_-citrate; pH 7.0) for 10 min. Membranes were then air-dried and baked at 80°C for 60 min to cross-link the transferred DNA. All membranes were stored at 4°C until hybridization.

### Hybridization and detection protocol

The treated nylon membranes were placed in hybridization glass bottles and pre-hybridized at 50°C in a hybridization oven for 1 h in hybridization buffer (50% v/v formamide; 5 X SSC; 1% v/v blocking reagent diluted in 0.1 M maleic acid and 0.15 M NaCl, pH 7.5; 0.1% N-lauroylsarcosine; 0.02% v/v SDS), as previously described [38]. Labeled probes were denatured at 97°C for 5 min, placed on ice, mixed with pre-warmed hybridization buffer (2 μl probe/ml buffer) and transferred in sterile tubes with the appropriate membrane. Hybridization buffers containing the labeled probes were stored at -20°C and reused several times, after denaturation at 65°C. Hybridization was carried out at 50°C for 3 h. Nylon membranes were then stringently washed in two washing solutions with constant agitation: 2 X SSC-0.1% SDS for 2 x 5 min at room temperature; 0.5 X SSC-0.1% SDS for 2 x 15 min at 67°C. The detection step was performed with the DIG Nucleic Acid Detection Kit (Roche), according to the manufacturer’s instructions. Briefly, unspecific binding sites were blocked with 1% blocking reagent for 30 min, successively the labeled probes were bound to anti-digoxigenin Fab-fragments conjugated to alkaline phosphatase for 30 min and, finally, antibody-binding was visualized by membrane incubation in NBT/BCIP solution until the color reaction was completed (30–60 min). Membranes were photographed, scanned and wet stored in plastic bags at 4°C for any further stripping and re-hybridization.

### Bacterial strain identification from mouse co-infection

From the plated serial dilutions used to determine the numbers of bacterial CFU recovered after mouse infection, plates with 200–600 single colonies were selected and examined by colony hybridization, as described above, in order to distinguish between colonies of *Y. enterocolitica* strains 8081 and WA-314. Membranes were either hybridized first with probe Hem_8081 or probe Col_WA, were stripped and re-hybridized with probe Col_WA or Hem_8081, respectively. The specificity of the probes was confirmed by analyzing plates containing either strain 8081 or strain WA-314.

### Colicin activity assay

Antibacterial activity of the *Y. enterocolitica* WA-314 colicin cluster was tested by the spot-on-lawn method for screening of inhibitory activity against *Y. enterocolitica* strain 8081 and *Escherichia coli* K12 strain MG1655, known to be susceptible to colicins. *Y. enterocolitica* strain WA-314 was grown overnight at 27°C in LB medium. From this liquid culture, two spots of 5 μl were made on solid LB medium; induction of colicin production was conducted adding mitomycin C in the solid LB medium at a final concentration of 0.5 μg/ml. The spotted WA-314 bacteria were incubated for 20 h at 27°C or 37°C. Cells were killed by exposure to 700 μl chloroform vapor for 10 min and dried for 20 min by aeration. The surface of the solid medium was overlaid with 7 ml of 0.7% soft agar containing 10 μl of an overnight culture of the indicator organism; *Y. enterocolitica* strain 8081-overlaid plates were grown at 27°C or 37°C and plates overlaid with *E. coli* K12 were grown at 37°C.

### Genome sequencing and comparison

A high-quality *Y. enterocolitica* strain WA-314 genome sequence was obtained in cooperation with BGI-Hongkong Co. (Hong Kong). High-throughput Illumina sequencing technology was used to construct 500-bp library with expected data of 500 Mb, and 6-kbp library with expected data of 250 Mb. Assembly of 15-bp short reads by the SOAPdenovo assembler resulted in 32x genome depth. Genome sequence was annotated by the RAST server [39] and tRNA identification was confirmed using tRNAscan-SE [40]. This Whole Genome Shotgun project has been deposited at DDBJ/EMBL/GenBank under the accession number AKKR00000000. The version described in this paper is the first version, AKKR01000000. Comparison with the *Y. enterocolitica* strain 8081 genome sequence [GenBank: AM286415 and AM286416 (plasmid)] [12] was performed using BRIG [41], SEED [42] and the progressive Mauve [43] algorithm with default settings and a 1,500 bp cutoff as the minimum LCB length. Orthologous proteins were determined considering a minimum of 50% of sequence similarity between bi-directional hit proteins. Specific genes and gene clusters were aligned with ClustalW [44] and manual homology searches were performed by BLAST analysis [45]. To identify protein similarity with characterized proteins and known functional domains, we searched the NCBI conserved domain database (CDD) [46] or the Pfam protein database [47].

### Phylogeny reconstruction

To infer *Yersinia enterocolitica* phylogeny, we selected 9 strains with available genome sequences and we chose *Y. pestis* strain CO92 as the outgroup; accession numbers are listed in Table 6. Phylogenetic trees were constructed using 5 housekeeping genes that were head-to-tail concatenated into a string of about 9200 bps for each strain: *glnA* (glutamine synthetase), *gyrA* and *gyrB* (DNA gyrase subunit A and B), *groEL* (60 kDa chaperonin) and *recA* (recombinase A). Protein sequences were aligned using Muscle [48] and the protein alignment was used as a model to create the DNA alignment with RevTrans [49]. Uninformative characters were removed using Gblocks [50] and phylogenies based on both DNA and protein alignments were reconstructed with Phylip [51] under a neighbor-joining model. A majority rule-consensus tree of 100 bootstrap replicates was also computed to evaluate node support. 

**Table 6:** Accession numbers of the genome sequences used in this study

**Organism**	**Accession number**
*Y. enterocolitica* WA-314, 1B/O:8	GenBank: AKKR01000000
*Y. enterocolitica* 8081, 1B/O:8	GenBank: AM286415, AM286416 (plasmid)
*Y. enterocolitica* a127/90, 1B/O:8	NCBI RefSeq: NC_004564 (plasmid)
*Y. enterocolitica* Y11, 4/O:3	GenBank: FR729477, FR745874 (plasmid)
*Y. enterocolitica* Y8265, 4/O:3	GenBank: CACU01000001-CACU01000014
*Y. enterocolitica* Y5307, 4/O:3	GenBank: CACV01000001-CACV01000018
*Y. enterocolitica* 105.5R(r), 3/O:9	GenBank: CP002246
*Y. enterocolitica* W22703, 2/O:9	NCBI RefSeq: NC_002120 (plasmid)
*Y. enterocolitica* Y5.27P, 3/O:5.27	GenBank: CACW01000001-CACW01000020
*Y. enterocolitica* NF-O, 1A/O:5	GenBank: CACY01000001-CACY01000097
*Y. enterocolitica* IP2222, 1A/O:36	GenBank: CACZ01000001-CACZ01000074
*Y. pestis* CO92	GenBank: AL590842

## Competing interests

The authors declare that they have no competing interests.

## Authors’contributions

DG designed and executed all experiments, carried out the genome comparison and bioinformatics analysis and drafted the manuscript. HB performed the major part of the mouse infection experiments, provided ideas for the study and participated in the composition of the manuscript. JH and AR supervised the study and helped to draft the manuscript. AR also conceived of the study and participated in its design. All authors read and approved the final manuscript.

## Supplementary Material

Additional file 1 **Colony hybridization experiment.** A: nylon membrane from the liver of mouse number 13 (13 L), probed with Hem_8081. B: nylon membrane from A stripped and re-probed with Col_WA. C: original LB-agar plate. D: modified and superimposed images from A and B. (PDF 146 kb) Click here for file

Additional file 2 **Assembly in scaffolds and accession numbers of the contigs of*Y. enterocolitica*strain WA-314.** Click here for file

Additional file 3 **Predicted prophages in*Y. enterocolitica*strain WA-314.** Excel table with prophage names, respective contigs, locus tags and encoded products. Click here for file

Additional file 4 **Genetic structure of prophages in*Y. enterocolitica*strain WA-314.** Annotation of selected genes is shown. YWA-1: possible degenerate P2-like prophage, 34.4 kbs. YWA-2: putative bacteriophage, 33 kbs. YWA-3: putative P2-like prophage, 11.7 kbs. YWA-4: putative P4-like prophage, 28 kbs. YWA-5: Mu-like prophage, 43.3 kbs. YWA-6: P4-like prophage, 14.6 kbs. YWA-7: putative defective prophage, 12.5 kbs. Click here for file
