# Supplementary figures and images for: Tracing genomic variations in two highly virulent Yersinia enterocolitica strains with unequal ability to compete for host colonization
Source: BMC Genomics. 2012 Sep 11;13:467. doi: 10.1186/1471-2164-13-467 (PMC3469391; doi:10.1186/1471-2164-13-467)

A

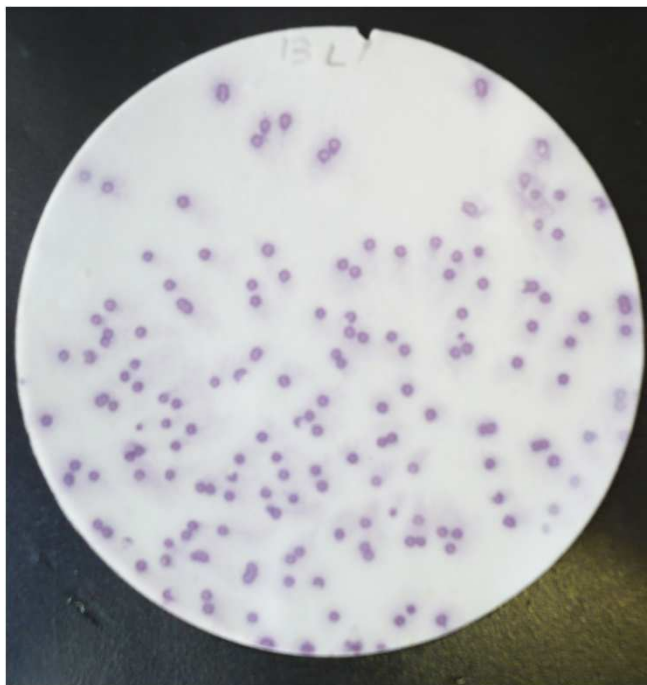

B

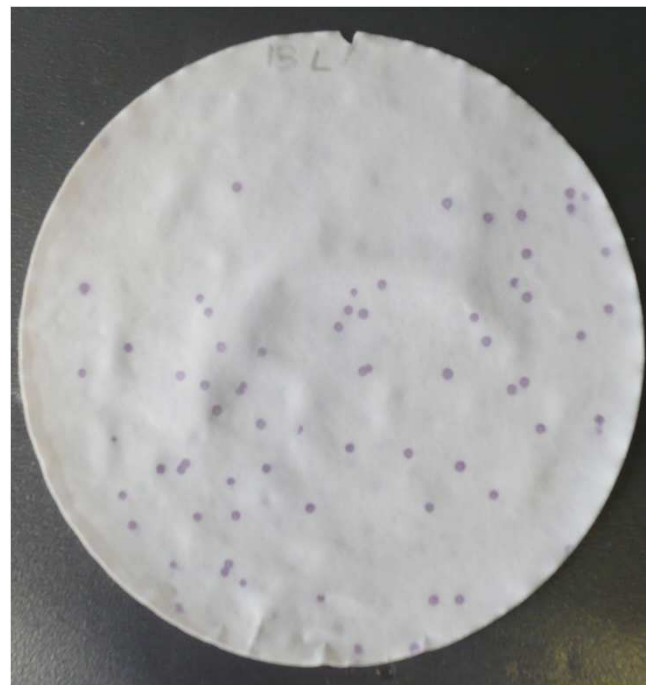

C

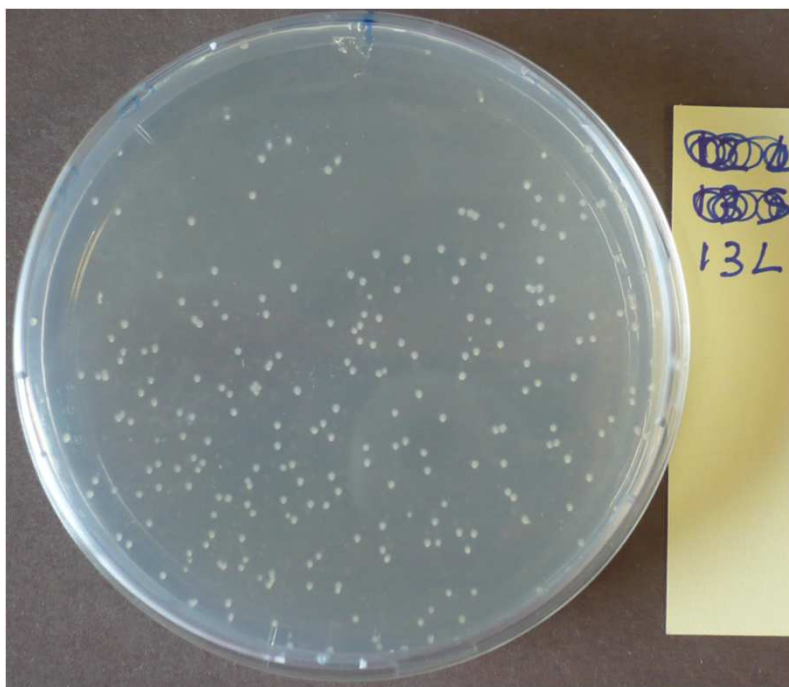

D

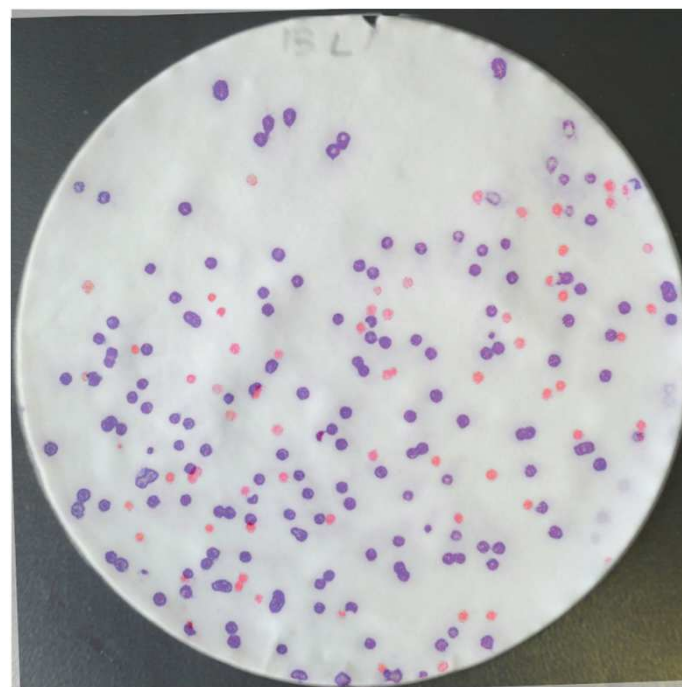

Supplement: Additional file 1 — Colony hybridization experiment. A: nylon membrane from the liver of mouse number 13 (13 L), probed with Hem_8081. B: nylon membrane from A stripped and re-probed with Col_WA. C: original LB-agar plate. D: modified and superimposed images from A and B. (PDF 146 kb) [file 1471-2164-13-467-S1.pdf]

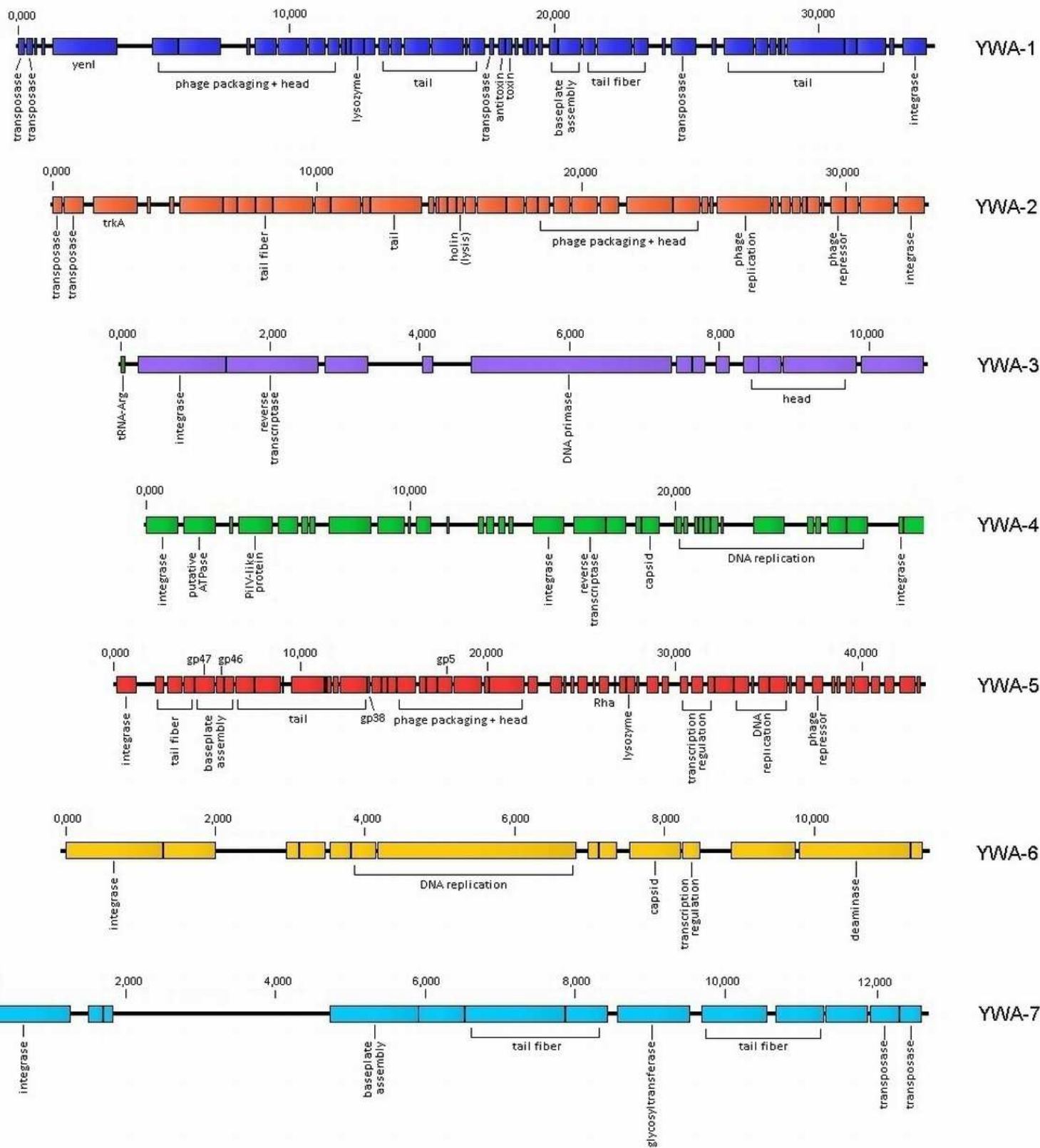

Supplement: Additional file 4 — Genetic structure of prophages inY. enterocoliticastrain WA-314. Annotation of selected genes is shown. YWA-1: possible degenerate P2-like prophage, 34.4 kbs. YWA-2: putative bacteriophage, 33 kbs. YWA-3: putative P2-like prophage, 11.7 kbs. YWA-4: putative P4-like prophage, 28 kbs. YWA-5: Mu-like prophage, 43.3 kbs. YWA-6: P4-like prophage, 14.6 kbs. YWA-7: putative defective prophage, 12.5 kbs. [file 1471-2164-13-467-S4.pdf]
